# Preparedness of emergency care providers to manage obstetric haemorrhage in KwaZulu-Natal province, South Africa: A mixed-methods study

**DOI:** 10.4102/jcmsa.v4i1.349

**Published:** 2026-06-18

**Authors:** Shaun Govender, Olive P. Khaliq, Tashlen Abel, Jagidesa Moodley

**Affiliations:** 1 KwaZulu-Natal College of Emergency Care, Durban, South Africa; 2 Women’s Health and HIV Research Group, Discipline of Obstetrics and Gynaecology, School of Medicine, College of Health Sciences, University of KwaZulu-Natal, Durban, South Africa; 3 Department of Paediatrics and Child Health, School of Clinical Medicine, Faculty of Health Sciences, University of the Free State, Bloemfontein, South Africa; 4 Discipline of Otorhinolaryngology and Head & Neck Surgery, College of Health Sciences, University of KwaZulu-Natal, Durban, South Africa; 5 Optics and Imaging Center, School of Health Sciences, College of Health Sciences, University of KwaZulu-Natal, Durban, South Africa

**Keywords:** emergency medical services, preparedness, emergency care providers, obstetric haemorrhage, KwaZulu-Natal province

## Abstract

**Background:**

Obstetric haemorrhage (OH), particularly postpartum haemorrhage, is a leading cause of maternal mortality in sub-Saharan Africa. Emergency medical services (EMS) play a critical role in early recognition, intervention and transport; however, evidence on EMS preparedness for OH remains limited. This study assessed the system-level preparedness of public-sector emergency care providers in KwaZulu-Natal (KZN) across four domains: administration, resources, training and response capacity.

**Methods:**

A concurrent convergent mixed-methods design was used. Quantitative data were collected using structured questionnaires (*n* = 417) and analysed descriptively. Qualitative data were obtained through semi-structured interviews (*n* = 10) and thematically analysed. Findings were integrated to identify convergence and divergence across data sources.

**Results:**

Key gaps were identified across all four domains. Administrative weaknesses included inconsistent policy implementation and limited supervisory oversight. Resource constraints reflected ambulance shortages and variable access to essential equipment. Training gaps included limited hands-on obstetric exposure and variable practical skills on scene. Response capacity was affected by the absence of maternal-specific surge planning.

**Conclusion:**

Emergency medical services in KZN play a central role in the prehospital management of OH but operate within significant system-level constraints. Strengthening governance, improving fleet and equipment availability, expanding practical obstetric training and integrating maternal health into surge and disaster planning may enhance preparedness in KZN and similar resource-limited settings.

**Contribution:**

This study provides evidence on EMS preparedness to manage obstetric haemorrhage, informing system strengthening and maternal emergency care planning.

## Introduction

Globally, a woman dies from maternal complications approximately every 2 min, with sub-Saharan Africa (SSA) bearing 70% of this burden.^1^ Obstetric haemorrhage (OH), described as bleeding from 20 weeks of gestation to the postpartum period,^2,3^ is one of these complications and remains the leading cause of maternal death worldwide.^4^ However, low- and middle-income countries (LMICs) such as South Africa (SA), are disproportionately impacted, largely due to ‘inequalities in health infrastructure and inequitable access to services’.^5,6^ Patients with OH, if not managed efficiently, may rapidly deteriorate leading to poor outcomes.^7^ Within SA, the KwaZulu-Natal (KZN) province reports the highest rates of maternal mortality attributable to OH, underscoring its significant local burden.^8^

In 2023, an estimated 260 000 maternal deaths occurred worldwide.^1^ The World Health Organization (WHO) noted that most of these women ‘could have survived pregnancy and childbirth if they had sufficient access to lifesaving care before, during, and after delivery’.^1^ The WHO has consistently recommended that emergency medical services (EMS) be integrated into district health systems to strengthen universal health coverage and reduce preventable maternal morbidity and mortality.^1,9^ As the first point of clinical contact for many women in rural and underserved areas, EMS plays a critical role in the early recognition, timely response, initial management and transport of obstetric emergencies such as OH. However, the ability of EMS systems to perform this role effectively depends heavily on their level of preparedness defined by functional administration (including referral systems), adequate resources, routine training and resource capacity to sustain prioritised emergency response during high-demand or surge events.^5,10^

In SA, existing literature describes a constrained EMS system with significant challenges.^5^ To strengthen maternal healthcare, and in response to a national strategy,^11^ EMS introduced dedicated obstetric ambulances to prioritise obstetric emergencies.^12^ Despite this, little is known about the preparedness of emergency care (EC) providers to manage these emergencies.

This article forms part of a broader mixed-methods study examining EC provider capacity to manage OH in the prehospital setting. While the larger study addressed multiple objectives, including providers’ knowledge, attitudes, and practices, those findings have been published separately.^13^ The present manuscript focuses specifically on the system-level preparedness component of that broader study.

Preparedness was conceptualised as the organisational and operational conditions that enable or constrain EC providers’ ability to effectively manage OH. The study therefore aimed to describe EC provider preparedness in KZN across four system domains: governance and administration, resources, training systems and response capacity.

## Research methods and design

### Study design and setting

A pragmatic, concurrent, convergent mixed-methods design was used to describe EC provider preparedness to manage OH in the prehospital setting. Quantitative data were collected through a cross-sectional survey, and qualitative data were generated through semi-structured interviews conducted during the same study period. Both strands were given equal priority and analysed independently prior to integration. Quantitative findings described the extent and distribution of preparedness indicators, while qualitative findings provided contextual insight into organisational, operational, and workforce factors influencing preparedness.

Preparedness was operationalised across four system-level domains: administration, encompassing governance and supervisory processes; resources, relating to the availability and functionality of ambulances and essential equipment; training, referring to access and participation in obstetric training programmes; and response capacity, reflecting system-level factors that contribute to delayed responses during high-demand periods. These domains were informed by the Integrated Healthcare Service Delivery Framework articulated in the Referral Policy for South African Health Services,^10^ which outlines structural and functional components required for effective emergency and referral systems. Survey items and interview questions were identified a priori and developed to align with these framework-informed domains.

The study was conducted in KZN, one of nine provinces in SA, covering an area of 94 361 km^2^ with an estimated population of 12.3 million, predominantly residing in rural areas.^14,15^ The province is administratively divided into 11 health districts, all of which are served by public-sector EMS.^16^

### Study population

#### Quantitative phase

Emergency care providers registered with the Health Professions Council of South Africa (HPCSA) and working in KZN’s public-sector EMSs formed the quantitative study population. In SA, these providers are registered across several categories that differ in training duration, academic level and clinical scope. For analytical purposes, these were grouped into three levels of care: basic life support (BLS), intermediate life support (ILS) and advanced life support (ALS) (Online Appendix 1).^17^

#### Qualitative phase

The qualitative study population comprised EC providers with ILS or ALS qualifications, selected for their higher clinical scope and system-level experience. Some qualitative participants had also completed the quantitative survey, while others were recruited independently based on their managerial or operational roles, to ensure inclusion of system-level perspectives not fully represented in the survey sample. The final sample comprised seven managerial participants and three frontline ambulance-based providers, enabling exploration of governance, resource allocation, training systems and operational response capacity from both organisational and frontline perspectives in the prehospital setting.

### Inclusion and exclusion criteria

#### Quantitative phase

Emergency care providers with BLS, ILS or ALS qualifications, who were registered with the HPCSA and employed in the public-sector EMS in KZN, were eligible for inclusion. Providers not working on ambulances at the time of data collection were excluded.

#### Qualitative phase

Emergency care providers with ILS or ALS qualifications were eligible for inclusion, if they held an active role in the management of obstetric emergencies and service-level planning for maternal emergencies. Providers not engaged in EMS management or ambulance-based care were excluded.

### Sampling strategy and sample size determination

#### Quantitative phase

Convenience sampling was used to recruit EC providers across all 11 health districts in KZN. This approach was selected to facilitate access to providers across operational shifts and geographically dispersed ambulance bases within the public-sector EMS. Providers were approached in person at ambulance bases during shift changes and were invited to participate voluntarily, if they met the inclusion criteria. As participation was dependent on availability during data collection, the sample may not fully represent providers unavailable due to operational demands.

Indicative sample size targets were calculated for each district using the Raosoft sample size calculator application (Raosoft Inc., Seattle, US),^18^ with a 95% confidence interval and a 5% margin of error, based on an estimated population of 2742 registered EC providers. This yielded a minimum required sample size of 338 participants to achieve representativeness at the provincial level. These calculations informed recruitment planning but did not constitute stratified sampling. Actual recruitment was dependent on provider availability during the data collection period. A total of 417 completed questionnaires were obtained, exceeding the minimum calculated requirement and thereby improving the precision of the estimates.

#### Qualitative phase

Purposive sampling was used to recruit eligible ILS and ALS providers meeting the predefined inclusion criteria. Participants were selected based on professional role, level of qualification (ILS or ALS), years of experience and involvement in either operational management or frontline ambulance-based care. Interviews were conducted iteratively, with concurrent analysis informing ongoing recruitment, until thematic saturation^19^ was reached. This occurred by the 10th interview, with no new preparedness related data identified.

### Data collection

#### Quantitative phase

A 60-item self-administered questionnaire, completed independently by participants in paper-based format, was utilised for data collection. The instrument assessed multiple constructs within the broader mixed-methods study, including knowledge, attitudes, practices, and system-level preparedness related to OH. For the purposes of this manuscript, only items pertaining to preparedness were analysed. These items aligned with the four predefined system-level domains described earlier.

Content and face validity of the questionnaire were established through review by EMS educators, managers, an obstetrician and a statistician. The tool was piloted with 16 EC providers, leading to minor revisions; pilot data were excluded. Questionnaires were completed anonymously. The principal investigator (PI) collected these data between July 2022 and August 2023 at central venues in each of KZN’s 11 districts, with participants representing multiple ambulance stations.

Completed questionnaires were transcribed manually into an electronic database by the PI. To ensure data accuracy, a random sample of completed questionnaires was cross-checked against the electronic dataset, and discrepancies were resolved by reference to the original questionnaires. The questionnaire is provided as an electronic supplementary file (Online Appendix 2).

#### Qualitative phase

Semi-structured interviews were conducted by the PI in private offices during district visits. Interviews were audio-recorded with participant consent and transcribed verbatim in Microsoft Word. The interview was guided by a structured set of questions designed to elicit participants’ perceptions of preparedness, focusing on governance, resources, training and response capacity during periods of increased demand.

### Data analysis

#### Quantitative phase

Quantitative data were analysed using SPSS version 29 (IBM Corp., Armonk, NY, US). Descriptive statistics were reported as frequencies and percentages. Each questionnaire item was analysed as a domain-specific preparedness indicator, representing measurable components of administrative processes, resource availability, training systems and response capacity. In this study, response capacity refers to the system’s ability to mobilise, coordinate, and sustain timely obstetric emergency responses under routine and surge conditions including disasters and critical incidents. The analysis focused on identifying patterns and variability in system-level preparedness.

#### Qualitative phase

Qualitative data were analysed using an inductive thematic analysis approach. Transcripts were imported into NVivo (Qualitative Software Research International) and coded without a predefined framework, allowing themes to develop directly from the data. Codes were iteratively reviewed and refined into sub-themes and overarching themes through constant comparison and discussion within the research team.

Following theme development, the themes were mapped onto the four predefined preparedness domains (administration, resources, training and response capacity) to enable structured presentation of system-level preparedness aligned with the study objectives. A detailed tabulation of themes, sub-themes, and illustrative participant quotations is provided in Online Appendix 3, to support transparency and analytic auditability.

Trustworthiness of the qualitative findings was addressed using established criteria. Credibility was enhanced through peer review of coding and the inclusion of direct participant quotations, to support analytic interpretations. Dependability was supported through maintenance of an audit trail documenting coding decisions and theme development. Confirmability was strengthened through reflexive discussions within the research team, to minimise individual analytic bias during interpretation.

### Synthesis of quantitative and qualitative results

A joint display was used to synthesise quantitative and qualitative findings across the four preparedness domains. Integration occurred primarily at the interpretive stage, whereby findings from both phases were examined side by side to identify areas of convergence and divergence. This approach informed the integrated interpretation of results and supported the development of contextually relevant recommendations.

### Ethical considerations

Ethics approval for the study was granted by the University of KwaZulu-Natal Biomedical Research Ethics Committee (BREC) (ref. no. BREC/00003780/2022) and the KZN Provincial Department of Health (ref. no. KZ_202205_008). These approvals covered the parent mixed-methods study, including use of the EMS database and data collected for this specific preparedness component. Participation was entirely voluntary, and written informed consent was obtained individually. All data were securely stored with password protection and accessible only to the PI.

## Results

### Participant characteristics

The demographic and professional characteristics of participants in the quantitative and qualitative phases are presented below:

- **Quantitative phase:** All 417 EC providers completed the questionnaires. Most were male (75.3%, *n* = 314), ≥ 40 years (66.4%, *n* = 277) and registered with the HPCSA for ≥ 5 years (72.2%, *n* = 301). By level of care, 35.0% (*n* = 146) were BLS providers, 59.7% (*n* = 249) ILS providers and 5.3% (*n* = 22) ALS providers.
- **Qualitative phase:** Ten participants were interviewed (60% male, *n* = 6). Ages ranged from 40 years to 61 years (median 52). By qualification, 60% (*n* = 6) were ALS and 40% (*n* = 4) ILS providers. By role, 70% (*n* = 7) were in EMS management (four ILS and three ALS), while 30% (*n* = 3) were ALS providers working on ambulances.

### Quantitative results

Survey questions assessed preparedness indicators across the four predefined domains:

- **Administration:** Most providers (98.1%, *n* = 409) reported conducting equipment and supply checks at the start of each shift. However, 46.5% (*n* = 194) indicated that vehicle checklists were never formally verified. Access to ALS clinical advisory support was inconsistent: 44.6% (*n* = 186) reported occasional access and 14.6% (*n* = 61) reported no access.
- **Training:** Significant gaps were identified in obstetric training exposure. A majority (74.3%, *n* = 310) reported not reviewing updated obstetric Clinical Practice Guidelines (CPGs). Similarly, most respondents lacked mandatory CPG training (80.1%, *n* = 334), simulation-based Essential Steps in the Management of Obstetric Emergencies (ESMOE)^20^ training (89.2%, *n* = 372), and training in the use of non-pneumatic anti-shock garments (NASG)^21^ (85.1%, *n* = 355). Among those offered training opportunities, 41% (*n* = 168) cited transport barriers as a limiting factor.
- **Resources:** Most participants reported access to oxygen (97.4%, *n* = 406), intravenous fluids (92.3%, *n* = 385) and sanitary pads (93.5%, *n* = 390). Access to monitoring equipment was more limited, with 77.5% (*n* = 323) reporting availability of blood pressure cuffs and 71.2% (*n* = 297) reporting pulse oximeters. Only 5.8% (*n* = 24) reported access to a NASG.
- **Response capacity:** Operational contributors to delayed response were frequently reported. Of 417 participants, 68.1% (*n* = 284) identified vehicle repairs as a source of delay, 44.6% (*n* = 186) cited equipment availability issues, and 31.9% (*n* = 133) reported call centre queries. Regarding shift handover, 28.1% (*n* = 117) noted delays during handover. Multiple responses were permitted for this domain; therefore, percentages represent the proportion of participants selecting each factor and do not total 100%.

### Qualitative results

Three overarching themes were constructed inductively from the interview data to describe EC providers’ preparedness to manage OH: (1) established measures for OH management; (2) current case approach and oversight; and (3) challenges and impact on EMS response. Themes and sub-themes were developed through iterative coding and constant comparison.

For coherence with the study aim, the themes were subsequently mapped onto the four predefined preparedness domains (administration, resources, training and response capacity) to enable structured presentation of system-level preparedness. A detailed tabulation of themes, sub-themes and illustrative participant quotations is provided in Online Appendix 3 to support transparency and analytic auditability.

#### Theme 1: Established measures for obstetric haemorrhage management

Participants described established EMS practices based on guidelines, dispatch protocols and dedicated ambulances. This theme reflects preparedness within the administrative and response capacity domains, with particular reference to dispatch protocols and ambulance allocation structures.

**Sub-theme 1.1: Emergency medical services guidelines and policies:** Formal policies directed that all obstetric cases be treated as red code priority. These policies were described as standardised and widely understood, suggesting the presence of formal governance mechanisms to support prioritisation of OH cases:

‘All maternity ambulance patients are red code priority cases according to established policies.’ (P4, 49 years old, male)

**Sub-theme 1.2: Dispatch protocols and ambulance allocation:** The call centre followed dispatch protocols to allocate ambulances. Dedicated obstetric ambulances were used when available; otherwise, general-use ambulances responded. Participants indicated that while protocols existed, their implementation was contingent on resource availability rather than clinical need alone:

‘If they have an obstetric vehicle available, the obstetric vehicle is dispatched. Alternatively, the first available ambulance is dispatched.’ (P3, 52 years old, male)

#### Theme 2: Current case approach and oversight

Participants discussed their approach to the management of OH, dispatch practices and quality assurance procedures. This theme reflects preparedness across the four domains, highlighting how formal policies are operationalised in routine practice.

**Sub-theme 2.1: Red code triage and prioritisation:** Obstetric emergencies, including OH, were triaged as red code (priority one) in line with WHO and national health guidelines. Participants generally described this approach as well established at the point of call taking, reflecting alignment between policy and dispatch intent:

‘Currently, we try to follow the WHO guidelines where we prioritise obstetric cases by making them red codes.’ (P3, 52 years old, male)

**Sub-theme 2.2: Dispatch driven by proximity rather than skill level:** Dispatch decisions were often based on proximity of the ambulance to the patient location, rather than EC provider skill level. This approach was described as necessary to minimise response times but was perceived to limit the ability to match clinical complexity with provider capability:

‘We just send the first available vehicle, regardless of qualification.’ (P6, 40 years old, male)

**Sub-theme 2.3: Feedback and quality assurance mechanisms:** Formal quality assurance initiatives were limited. Patient Report Form (PRF) audits were usually complaint-driven, with little oversight beyond response time tracking. Participants indicated that the absence of routine clinical audit and feedback mechanisms constrained opportunities for learning, accountability, and system-level improvement:

‘Yes, we do PRF checks and audits … but only if there’s a complaint or something flagged.’ (P8, 43 years old, male)

‘None. The only tool that EMS uses currently as a guideline is response times ….’ (P3, 52 years old, male)

#### Theme 3: Challenges and impact on emergency medical services response to obstetric emergencies

Participants identified systemic and operational challenges, adverse outcomes for patients and limited preparedness for critical incidents and high-demand events. This theme reflects gaps across the administrative, resource, training and response capacity, illustrating how system constraints affect the management of obstetric emergencies in practice.

**Sub-theme 3.1: Inadequate policy dissemination and implementation:** Policies existed but were poorly communicated and inconsistently applied. Participants described reliance on informal communication rather than structured implementation mechanisms, limiting consistency in practice:

‘It was a verbal instruction to treat these as red codes, but there’s no formal system.’ (P6, 40 years old, male)

**Sub-theme 3.2: Shortage of ambulances and delayed response:** Ambulance shortages frequently caused long delays, even for priority (red code) cases. These delays were described as operational rather than clinical in origin, highlighting the impact of resource constraints on response capacity:

‘Sometimes we wait three, four hours … and by the time we get there, it’s too late.’ (P2, 46 years old, female)

‘Yes. Shortage of ambulances, they do have impact in terms of response time.’ (P8, 43 years old, male)

**Sub-theme 3.3: Critical incidents and patient harm:** Poor communication and delayed response sometimes led to adverse outcomes. Participants linked these incidents to breakdowns in interfacility communication and escalation processes during obstetric emergencies:

‘The clinic nurse told us the delivery was done and to take another patient, but the woman started bleeding again. She arrested on the way to hospital.’ (P9, 55 years old, female)

**Sub-theme 3.4: Surge and disaster planning gaps:** Participants described the absence of critical event and disaster plans, let alone accommodating obstetric emergencies. Ambulance shortages during high-demand events were reported to constrain EMS capacity, with no contingency arrangements to protect obstetric responses when multiple emergencies occurred simultaneously. Quantitative findings relating to delayed response and operational constraints support these qualitative accounts of critical incident unpreparedness and system strain, suggesting limited system response capacity during high-demand events:

‘There is no plan in existence for major incidents and disasters.’ (P1, 61 years old, male)

‘If all vehicles are attending a disaster, no ambulances would be available for other patients.’ (P4, 49 years old, male)

**Sub-theme 3.5: Gaps in training systems, delivery and workforce readiness:** Participants described training-related challenges including limited access to refresher and advanced obstetric training, a predominance of BLS-qualified staff and variable engagement with upskilling. Reduced hands-on training opportunities and limited training for call centre personnel were perceived to weaken preparedness and escalation during obstetric emergencies.

Quantitative evidence of limited guideline review and training exposure aligns with these qualitative accounts of fragmented training systems and workforce readiness gaps:

‘If they don’t have the mindset that an obstetric is an emergency … they’re not going to be doing it.’ (P2, 46 years old, female)

‘There should be a special course of refresher for our practitioners for these cases.’ (P4, 49 years old, male)

### Integration of quantitative and qualitative findings

Quantitative and qualitative findings were integrated using a joint display (Table 1) to compare results across the four preparedness domains. Integration occurred primarily at the interpretive stage, whereby findings from both phases were examined side by side to identify areas of convergence and divergence. This approach informed the integrated interpretation of results and supported the development of contextually relevant recommendations.

**TABLE 1:** Integrated presentation of quantitative and qualitative findings across preparedness domains.

Preparedness domain	Qualitative findings	Quantitative findings
Administration	Formal EMS policies designated obstetric cases as red code priority. Dispatch protocols guided ambulance allocation for obstetric emergencies. Dedicated obstetric ambulances were used when available, with fallback to general ambulances.	Providers reported routinely checking vehicles and equipment at the start of shifts. Supervisor verification of vehicle checklists was inconsistently reported. Access to clinical advice (ALS or senior support) varied.
	Integrated interpretation Although formal policies and dispatch protocols for obstetric emergencies were reportedly in place, their inconsistent implementation and limited oversight suggest a gap between documented governance structures and actual operational practice.	
Training	Limited access to structured refresher and obstetric training was described. A predominance of BLS-qualified staff and variable engagement with upskilling were reported. ‘Hands-on’ obstetric training was reduced with increased reliance on online formats. Gaps in training for call centre personnel were perceived to affect decision-making and escalation.	Most providers reported not reviewing updated obstetric CPGs. Mandatory training on obstetric CPGs was not routinely received. Simulation-based obstetric emergency training was infrequently reported. Training attendance was limited by operational constraints such as transport availability.
	Integrated interpretation Convergent findings across both strands indicate inconsistent access to structured obstetric training and limited opportunities for practical skill reinforcement, contributing to variable provider confidence and preparedness for time-critical emergencies.	
Resources	Shortages of ambulances were described as frequent and system-wide. Resource constraints were identified as a major contributor to delayed responses, including for red code obstetric cases. Limited availability of vehicles during concurrent incidents reduced EMS response capacity. Absence of contingency planning for resource surges was described as constraining system resilience during obstetric emergencies.	Providers reported access to basic clinical resources such as oxygen, intravenous fluids and sanitary pads. Access to monitoring equipment (e.g. blood pressure devices, pulse oximetry) was variable. Limited access to specialised obstetric equipment, including non-pneumatic anti-shock garments, was reported. Vehicle unavailability and repairs were commonly reported as causes of response delays.
	Integrated interpretation Findings across quantitative and qualitative data demonstrate that resource limitations, particularly vehicle availability and essential equipment systematically constrain timely emergency response and weaken operational readiness for obstetric emergencies.	
Response capacity	Providers described delayed or inappropriate EMS responses during time-critical obstetric emergencies, reflecting periods of increased service demand and dispatch system limitations. Critical incidents, including maternal deterioration and death, were linked to delayed ambulance arrival and communication breakdowns across facilities and EMS. Participants reported the absence of formal disaster or major incident plans specific to obstetric emergencies. Concurrent high-acuity events were described as overwhelming available ambulance resources, compromising sustained obstetric emergency response.	Providers reported multiple causes of delays during obstetric emergencies, including vehicle repairs, equipment availability and call centre processes. Delays related to operational factors (e.g. vehicle availability, shift handovers) were commonly reported. Emergency response capacity indicators demonstrated variability across districts and provider levels, indicating inconsistent operational readiness for time-critical obstetric cases.
	Integrated interpretation Integrated findings suggest that periods of increased service demand, coupled with coordination and communication challenges, compromise EMS capacity to sustain timely and prioritised response to obstetric emergencies.	

EMS, emergency medical services; ALS, advanced life support; BLS, basic life support; CPG, clinical practice guideline.

## Discussion

Using a concurrent convergent mixed-methods approach, this study examined preparedness for OH across four domains: administration, resources, training systems and response capacity. Integration of quantitative and qualitative findings provided a systems-level understanding of organisational strengths and operational constraints within the public-sector EMS in KZN. Across domains, findings indicate that while formal structures exist, their implementation and operationalisation remain inconsistent, particularly under conditions of system strain.

Administrative preparedness and oversight were limited, falling short of standards outlined in the SA Health Service Delivery Platform.^10^ Interviews with EC providers indicated that triage of obstetric emergencies was frequently communicated verbally, with frontline staff reporting that despite managers indicating that formal documentation existed:

‘… there’s nothing we’ve seen in writing’ (P1, 61 years old, male)

This disconnect suggests variability in how administrative processes were communicated and operationalised at the frontline.

Integrated findings indicated that ambulances were dispatched to obstetric emergencies based on proximity rather than crew capability, contrary to existing policies and procedures,^22^ suggesting weaknesses in policy implementation and dispatch governance. Survey findings showed that routine operational checks were commonly performed; however, supervisory audit and clinical quality assurance mechanisms were inconsistently applied. Qualitative interviews reinforced these findings, with one provider noting:

‘… we’re not effective enough … we haven’t got that quality assurance, that quality control sort of mechanism …’ (P5, 43 years old, female)

Together, quantitative and qualitative evidence indicate weaknesses in accountability and monitoring systems, with frontline providers managing OH cases under limited structured supervision. While governance frameworks and clinical guidelines appear to be well developed at national and provincial levels, strengthening implementation and performance monitoring mechanisms may be necessary to ensure consistent operational application within the EMS system.

Similar challenges have been identified in rural EMS systems internationally. In the United States (US), Hansen et al.^23^ reported absence of standardised maternal protocols, reliance on informal communication, and reactive supervision similar to this study. These patterns suggest that suboptimal administrative functioning can impair EMS response well before clinical care begins, thereby placing already vulnerable maternal patients at greater risk. Strengthening administrative preparedness will require formal enforcement of documented triage and dispatch protocols, routine supervisory audit of obstetric case management and improved visibility of written policies at station level. Clear accountability structures and active clinical oversight may reduce variation between governance intent and frontline implementation.

Resource preparedness emerged as a significant constraint within this study, with both quantitative and qualitative findings highlighting shortages and operational delays affecting timely prehospital care. Survey data indicated that while basic monitoring equipment was generally available, access to specialised obstetric resources such as NASGs was limited. Qualitative interviews reinforced these findings, particularly among ILS providers, who reported being first on scene:

‘… standing there waiting, knowing what’s needed but not having the equipment’ (P9, 55 years old, female)

Integrated findings further revealed limitations in fleet capacity and availability. Dedicated obstetric ambulances were scarce, and general-purpose ambulances were frequently unavailable due to mechanical failures and prolonged repair times. One participant described:

‘… the ambulance is booked off and then it just sits for weeks before it’s fixed’ (P1, 61 years old, male)

This reflects inefficiencies in fleet maintenance systems. Variations in reported equipment availability across districts suggest differences in district practices and supply chain functioning. Collectively, these findings indicate that structural resource constraints reduce operational readiness and contribute to delayed emergency response.

Similar constraints have been reported in another study,^23^ where EC providers identified ambulance unavailability, distance, interfacility delays and poor communication as barriers to response. Findings from a related retrospective cross-sectional analysis conducted as part of the same broader study further demonstrated prolonged response times and interfacility transfer delays in pregnancy-associated haemorrhage cases in KZN.^24^ In SSA, ambulance breakdowns and shortages are well-documented contributors to maternal mortality.^25^ Together, this body of evidence underscores how resource and system constraints compromise prehospital maternal EC.

For EC providers, these limitations translate into arriving at emergencies without essential tools, increasing clinical risk. Such delays may compromise timely intervention, particularly in OH cases where rapid response and stabilisation are critical.^2^ Strengthening resource preparedness may require prioritised procurement and equitable distribution of essential obstetric equipment, including NASGs, alongside improved fleet maintenance systems to reduce prolonged ambulance downtime. Routine district-level monitoring of equipment availability and vehicle functionality may further enhance operational readiness.

Training preparedness emerged as a system-level limitation within this study, with both quantitative and qualitative findings indicating restricted access to structured obstetric training, limited opportunities for skills refreshment and inconsistent implementation of training programmes.

Survey findings showed that most respondents had not completed in-service obstetric training, simulation-based programmes such as ESMOE or NASG training, despite their inclusion in CPGs. Qualitative interviews contextualised these gaps, with one provider reporting:

‘… we are still having the challenge as we don’t have a district trainer in our sub-district’ (P4, 49 years old, male)

This highlights structural constraints in training delivery.

Basic life support and ILS providers, who are frequently first on scene in OH cases, operate within limited scopes of practice and reported insufficient access to structured obstetric training, affecting early recognition, escalation and initial management. While the shift to online learning during the coronavirus disease 2019 (COVID-19) pandemic enabled continued theoretical instruction, the reduced emphasis on practical, skills-based training limited preparedness for time-critical obstetric emergencies. Reinforcing convergence between quantitative training deficits and qualitative accounts of reduced practical exposure, one participant noted:

‘After COVID, everybody’s got into this mode of doing training online.’ (P1, 61 years old, male)

Across both strands, findings suggest that inconsistent access to ‘hands-on’ obstetric training contributes to variable system readiness during maternal emergencies. These findings are consistent with international literature. Ameh et al.^26^ reported that ‘hands-on’ obstetric training improves provider knowledge retention and clinical performance. The recurrence of similar gaps across under-resourced EMS systems suggests that simulation and structured training are often deprioritised in constrained settings. Without accessible, practical continuous professional development (CPD) opportunities, EC providers may struggle to meet the required standard of care. Embedding structured, simulation-based obstetric training within routine CPD platforms, alongside protected time for skills refreshment, may strengthen early recognition, escalation, and on scene management of obstetric emergencies, particularly among BLS and ILS providers.

Response capacity preparedness in this study revealed substantial vulnerabilities in the EMS system’s ability to sustain obstetric emergency response during high-demand events. Qualitative interviews described response delays of up to 5 h, with ambulance unavailability frequently cited as the primary barrier. One participant illustrated how periods of increased service demand disproportionately affect obstetric emergencies, by noting that due to these delays:

‘… we often have patients delivering on scene.’ (P9, 55 years old, female)

Many EC providers reported being unaware of any formal critical event or disaster response plan, particularly for obstetric emergencies, suggesting gaps in structured surge planning required to maintain service continuity during high-demand events. Quantitative findings reinforced these concerns, identifying multiple contributors to delays, including ambulance repairs, prolonged handovers, call centre inefficiencies and unclear communication from facilities. These pressures, which persisted during the COVID-19 pandemic, point to limited system-wide coordination and the absence of clearly operationalised surge protocols.

Respondents described adverse maternal outcomes associated with delayed or fragmented responses, underscoring convergence between quantitative and qualitative strands in relation to response capacity preparedness. Similar patterns have been reported elsewhere,^23^ where the absence of coordinated, area-specific planning resulted in inappropriate referrals, fragmented communication and adverse outcomes. In KZN, the absence of maternal-specific critical event or surge protocols represents a significant system-level vulnerability.^5,24^

Integrated findings suggest that concurrent high-demand events compromise the system’s ability to maintain timely and prioritised obstetric emergency response. Importantly, response capacity did not operate independently; administrative weaknesses, resource shortages and training gaps collectively constrained surge readiness.

Development of maternal-specific surge protocols, embedded within district EMS operational plans, may strengthen response capacity by improving prioritisation, coordination and escalation during high-demand events. Clear escalation pathways and structured communication frameworks between dispatch centres, EC providers and referral facilities are essential to sustaining obstetric emergency response during periods of increased service demand.

In summary, this study identified four key findings affecting EC provider preparedness in managing OH: (1) gaps in policy implementation and supervisory accountability; (2) ambulance shortages and inconsistent access to essential equipment and medical supplies; (3) limited hands-on obstetric training and insufficient practical skills on scene; and (4) absence of formal surge planning to sustain response capacity during high-demand events. These interconnected system-level weaknesses compromise the timely and effective response to maternal emergencies.

This study has several limitations. Conducted in KZN, the findings may not be generalisable to other regions or LMIC settings. The use of a retrospective, self-reported paper-based survey may have introduced recall bias. In addition, the instrument was not formally validated, and categorisation by qualification may have obscured variation in training exposure and clinical practice. The predominance of managers in the qualitative sample may have influenced findings toward system-level perspectives.

This manuscript forms part of a broader mixed-methods study; however, the analyses presented here address distinct system-level preparedness objectives. Strengths include broad district coverage and a mixed-methods design that enabled triangulation across data sources. A total of 417 EC providers participated, representing approximately 15% of the eligible provincial workforce (*n* = 2742), enhancing confidence in the breadth of quantitative representation across districts. Although the preparedness survey contributed to a broader dataset used in a separate publication, the findings presented here are interpreted specifically in relation to system-level preparedness rather than individual knowledge, attitudes or practices.

## Conclusion

Obstetric haemorrhage remains a major threat to maternal health in KZN, compounded by gaps in EMS preparedness. This study identified weaknesses across four interrelated preparedness domains: administration, resources, training and response capacity. Strengthening these domains will require coordinated, system-level action, including improved governance and accountability mechanisms, structured and practical obstetric and NASG training embedded within continuing professional development platforms, and enhanced fleet maintenance and equitable distribution of essential equipment. Addressing these system-level gaps may strengthen prehospital management of OH. Future research should evaluate the effectiveness of targeted preparedness interventions on maternal emergency outcomes within EMS systems.
